# Renal and endothelial biomarkers in Chagas disease in the Brazilian Amazon region: Early indicators of kidney injury and disease progression

**DOI:** 10.1371/journal.pone.0353749

**Published:** 2026-07-17

**Authors:** Alba Regina Jorge Brandão, Jorge Augusto de Oliveira Guerra, Jessica Vanina Ortiz, Débora Raysa Teixeira de Sousa, Gabriela Maciel Alencar, Nádelly Karoline Martins Derze, João Victor Campelo de Queiroz, Joana Bader Sadala Brandão, Lara Isabelli Oliveira da Silva, Cássia Camila de Oliveira Araújo, Silvia Cassia Brandão Justiniano, Lucely Paiva Rodrigues da Silva, Elsa Isela Moctezuma-Guevara, Susan Smith-Doria, Karla Cristina Silva Petruccelli, Paula Rita Leite da Silva, Mônica Regina Hosannah da Silva e Silva, Kátia do Nascimento Couceiro, Gdayllon Cavalcante Meneses, Letícia Machado de Araújo, Elizabeth de Francesco Daher, Alice Maria Costa Martins, João Marcos Bemfica Barbosa Ferreira, Geraldo Bezerra da Silva Júnior, Maria das Graças Vale Barbosa Guerra

**Affiliations:** 1 Universidade do Estado do Amazonas, Manaus, Amazonas, Brasil; 2 Universidade Federal do Amazonas, Manaus, Amazonas, Brasil; 3 Fundação de Medicina Tropical Doutor Heitor Vieira Dourado, Manaus, Amazonas, Brasil; 4 Universidade Federal do Ceará, Fortaleza, Ceará, Brasil; 5 Universidade de Fortaleza, Fortaleza, Ceará, Brasil; 6 Fundação Hospitalar Alfredo da Matta, Manaus, Amazonas, Brasil; Oswaldo Cruz Foundation, BRAZIL

## Abstract

Chagas disease (CD) has impact on Amazon public health, where oral transmission is frequently related to acute cases. This peculiarity influences clinical severity and immunological responses, highlighting the need to investigate novel biomarkers for early detection of renal and endothelial dysfunctions, since conventional methods are limited in identifying subclinical renal injury. The aim of this study is to investigate the expression of biomarkers of renal and endothelial injury in acute and chronic CD in the Brazilian Amazon. A cross-sectional study was conducted between 2021 and 2023 at the Fundação de Medicina Tropical Doutor Heitor Vieira Dourado (FMTHVD), Manaus, Amazonas, Brazil. Seventy-eight native Amazonian patients diagnosed with CD were evaluated and grouped by disease clinical phase: G1a (acute pre-treatment), G1b (acute post-treatment), G2a (chronic indeterminate), and G2b (chronic cardiac). Blood and urine levels of SYN-1, ANG-2, MCP-1, and NGAL were quantified using ELISA test and correlated with creatinine, urea, proteinuria, and glomerular filtration rate (GFR). Biomarkers were elevated across CD phases, despite routine renal function parameters remaining within normal ranges. Urinary NGAL and MCP-1 levels were significantly elevated in G1, reflecting early renal inflammation. SYN-1 was elevated in patient groups compared with controls, indicating early endothelial damage, while ANG-2 showed high variability with limited subgroup discrimination in G2, suggesting progressive endothelial dysfunction. Overall, G1, especially G1b, exhibited a more severe inflammatory and tissue injury profile, whereas G2 groups were similar to controls. Conventional markers did not correlate with the biomarkers, suggesting their sensitivity in detecting early subclinical injury. The novel biomarkers studied showed an association with different phases of CD and signs of endothelial and renal dysfunction, suggesting potential to aid in the detection of subclinical changes related to the disease.

## Introduction

Chagas disease (CD) is one of the main neglected tropical diseases, with a significant impact on public health, especially in Latin America [1]. This disease can manifest in two clinical phases (acute and chronic) following contact through different routes with the infective form of *Trypanosoma cruzi* [2]. After infection by *T. cruzi*, a cascade of immune responses is triggered, involving inflammation and damage to multiple organs, including the cardiovascular system and the kidneys [3].

During the acute phase of CD, there is rapid multiplication of the parasite and a strong inflammatory response. This leads to intense cellular damage and tissue fibrosis compared to later stages [4,5]. In the chronic phase especially in areas with vector transmission, greater severity is observed with a high prevalence of heart failure and arrhythmias, which are responsible for significant morbidity and mortality [6–8].

Experimental studies show renal lesions in CD involve inflammation including functional and structural changes resulting from systemic inflammation, activation of the renin-angiotensin-aldosterone system (RAAS), and possible direct infection of renal tissue by the *T. cruzi* parasite [4,5]. The study of renal involvement in humans with CD emerges as a relevant aspect of the pathology, due to the interaction between immunological, inflammatory, and autoimmune responses. Furthermore, reduced renal blood flow, activation of the RAAS, and endothelial alterations contribute to the development of renal failure, which is often silent and underdiagnosed [9–11].

Conventional tests like creatinine and urea have limitations in the early detection of kidney damage [12]. The introduction of novel biomarkers, such as Neutrophil Gelatinase-Associated Lipocalin (NGAL), Syndecan-1 (SYN-1), Angiotensin II (ANG-2), and Monocyte Chemoattractant Protein-1 (MCP-1), has shown potential for identifying renal and endothelial alterations in tropical diseases. SYN-1 indicates tissue shedding, ANG-2 relates to inflammation, MCP-1 attracts immune cells, and NGAL signals tubular injury and cardiovascular severity. These markers could enable earlier diagnosis and personalized treatment, improving outcomes in regions with limited diagnostic options [13–22].

In the Brazilian Amazon, CD mainly spreads via oral transmission particularly through açaí contaminated with *T. cruzi*, leading to frequent acute outbreaks unlike in regions where vector-borne transmission predominates [6,23]. This epidemiological peculiarity contributes to the predominance of acute phase cases of the disease, characterized by clinical manifestations such as fever, headache, myalgia, facial edema, and cardiac dysfunction. The high prevalence of oral transmission together with a difficulty of accessing healthcare services, underdiagnosis, and failure in clinical follow-up after treatment makes the Brazilian Amazon a region requiring special attention [23,24]. In this region, between 2000 and 2025, around 3,800 acute CD cases were reported with most occurring in Pará, Brazil [25], where early detection occurs through the malaria surveillance network [26,27].

Furthermore, the impact of kidney impairment on patient prognosis remains an area scarcely explored within the context of Amazonian CD. Though known that renal injury can negatively influence outcomes, the incidence, and detailed characterization of such renal involvement in CD remains poorly documented [4]. In this context, our study aimed to investigate the expression and clinical relevance of non-traditional biomarkers of renal injury and endothelial activation—SYN-1, ANG-2, MCP-1, and NGAL—in patients with CD in the Brazilian Amazon region, with the goal of early detection of renal and vascular dysfunctions and improving clinical management of these patients.

## Methods

### Study design, setting and patient selection

This was a cross-sectional study performed at Fundação de Medicina Tropical Doutor Heitor Vieira Dourado (FMT-HVD), a referral center for tropical and infectious diseases in the state of Amazonas, northern Brazil. The study population included patients with CD in the acute and chronic phases, native from the Brazilian Amazon.

Recruitment took place between July 2021 and July 2023 at the FMT-HVD Chagas Disease Outpatient Clinic. Here patients were grouped according to the stage of the disease. Group 1 – Acute phase: encompassing patients diagnosed through the malaria surveillance network in the Amazon, through thick blood smear exams, subdivided into: G1a (pre-treatment, at the time of diagnosis), G1b (post-treatment). Group 2 – Chronic phase: involving patients referred from cardiology clinics, blood donation and survey services, with diagnosis confirmed by serology, subdivided into: G2a (chronic indeterminate) and G2b (chronic cardiac). All patients in this group were treated with benznidazole for 60 days.

Inclusion criteria were individuals with confirmed diagnosis of acute or chronic CD, of either sex, over 18 years of age and native to the Brazilian Legal Amazon were included. Group 1 – patients with positive parasitological diagnosis in acute phase, Group 2 – patients with reactive serology confirmed by at least two different diagnostic tests based on distinct methodologies. To establish baseline urinary biomarker levels, a control group consisting of 20 healthy individuals with non-reactive serology for CD, paired by age and sex, recruited at the Renal Diseases Center (CDR) in Manaus, Brazil was also included.

Patients that were pregnant, living or traveling for more than 3 months to regions outside the Brazilian Legal Amazon, with a history of chronic kidney disease, organ transplantation, serious cardiovascular diseases (such as heart failure or thrombosis), or using non-steroidal anti-inflammatory drugs (NSAIDs) were excluded.

### Ethical considerations

The Research Ethics Committee of the Fundação de Medicina Tropical Doutor Heitor Vieira Dourado approved this study under the CAAE number: 45886521.0.0000.0005, in accordance with Resolution No. 466/12 of the National Health Council of Brazil. Patients diagnosed with CD as well all individuals in the control group, who participated in the study provided a signed written informed consent and completed a standardized questionnaire.

### Biological samples

Biological sample collection and laboratory analysis – Serum samples (4mL) were obtained in sterile tubes with separating gel (BD Vacutainer® Serum Tubes), urine (10mL) in specific bottles and whole blood (10mL) in tubes with EDTA (ethylenediaminetetraacetic acid) (BD Vacutainer® Serum Tubes). The biological samples were stored in aliquots at −20ºC at Unidade de Entomologia Nelson Ferreira Fé (UENFF) at FMT-HVD, until use.

A complete blood count, biochemistry blood test (albumin, urea, creatinine, CPK, electrolytes), routine urine test and a 24-hour proteinuria were performed at the FMT-HVD Clinical Laboratory in the routine outpatient follow-up protocol. All patients underwent a kidney ultrasound, performed in a private clinic.

### Biomarker assay

The quantification of biomarkers was performed using the ELISA (Enzyme-Linked Immunosorbent Assay) test, utilizing specific aliquots of serum and urine collected from patients at different time points. Commercial kits with specific antibodies for the target biomarkers were acquired: urinary NGAL (R & D Systems, ref: DY1757), urinary MCP-1 (R & D Systems, ref: DY279), angiopoietin-2 (R & D Systems, ref: DY923), and syndecan-1 (Abcam, ref: ab308538). The concentrations of urinary NGAL and MCP-1 were normalized by urinary creatinine from the same sample.

The methodology followed standardized protocols for the sandwich-type immunoenzymatic assay. Initially, 96-well plates were sensitized with a specific capture antibody then incubated for a predetermined time. After blocking to prevent nonspecific binding, urine and serum samples were added along with the standard curve for quantification. A detection antibody conjugated to an enzyme was then added, followed by the addition of the chromogenic substrate. The reaction was stopped by adding acid, and the absorbance was read in a spectrophotometer at 450 nm.

Samples with biomarker concentrations below the assay detection limit were considered undetectable. Because these values do not represent true zero concentrations and their exact levels cannot be reliably determined, samples with undetectable results were excluded from quantitative analyses and graphical representation. The experiments were conducted at the Pharmaceutical Bioprospecting and Clinical Biochemistry Laboratory (LBFBC) of the Federal University of Ceará.

### *T. cruzi* genetic analysis

Complementarily, *T. cruzi* DTUs were obtained from a database of Dr. João Macias Frade Chagas disease research group. These strains were identified from blood samples from patients, using biomolecular techniques, according to the protocol used in the FMTHVD entomology laboratory [5] that consists of using *T. cruzi* blood cultures from patients for parasite DNA extraction. The DNA extraction from a peripheral blood sample (buffy coat) was performed following the PureLink™ Genomic DNA Mini kit protocol (Invitrogen, Life Technologies, California, USA). This was followed by performing the polymerase chain reaction (PCR) and its product was purified and submitted to sequencing using the ABI 3130 DNA sequencer (Applied Biosystems, https://www.thermofisher. com), following the Big Dye Terminator v.3.1 Cycle Sequencing Kit protocol (Applied Biosystems).

### Evaluation of cardiac involvement

The assessment of alterations in the electrocardiogram followed the guidelines established by the Brazilian Society of Cardiology regarding the diagnosis and treatment of patients with Chronic Cardiopathy due to Chagas Disease (CCDC). Criteria used for cardiac alterations: a) ECG outside normal limits; b) 24-hour Holter — Ectopic activity (Sporadic — > 200/24 hours; Mild — between 1 and 3% of the QRS number; Moderate — between 3% and 10% of the QRS number; Severe — between 10% and 30% of the QRS number; Very severe — above 30% of the QRS number); c) Echocardiogram: Left Ventricular Ejection Fraction (LVEF) ≤ 50%; increased size of the right and left cardiac chambers.

### Statistical analysis

Data normality was assessed using Shapiro-Wilk test. Continuous parametric variables (creatinine, urea and eGFR-Cr serum levels) were reported as means ± standard deviation (SD) and were compared across groups (Control, G1a, G1b, G2a, and G2b) using one-way analysis of variance (ANOVA). Continuous non-parametric variables (kidney injury and endothelial injury biomarkers) were reported as medians and interquartile ranges (IQR). Comparisons between two groups (with and without comorbidities) were performed using the Mann–Whitney U test. For comparisons across multiple clinical groups (control, G1a, G1b, G2a and G2b), the Kruskal-Wallis rank-sum test was employed, followed by Dunn’s post-hoc test for pairwise comparisons between the control and each clinical group. Biomarker distributions were visualized using violin plots overlaid with boxplots and individual data points. Categorical data (sex, case type, *T. cruzi* DTU, comorbidities, eGFR and cardiac alterations) were expressed as frequencies or proportions and were analyzed using chi-squared test or Fisher’s exact test, as appropriate. Correlations between renal biomarkers, endothelial biomarkers, and proteinuria were evaluated using Spearman’s rank correlation coefficient (ρ). All statistical analyses were performed using Stata v.14 (Stata Corp., USA), and p-value <0.05 was considered statistically significant.

## Results

### Socio-epidemiological and clinical laboratory aspects

The study included 78 patients, with a mean age of 43.5 ± 16.4 years with 43(55.1%) being male patients. The majority, 73(93.6%), came from 14 municipalities in the state of Amazonas; and 58(74.4%) were infected with *T. cruzi* during outbreaks due to oral transmission. Patient stratification by groups and disease duration, revealed 67(85.9%) patients were in G1, most of whom, 53 (67.9%), were in G1b (post-treatment). The average clinical follow-up time was 5.9 ± 5.2 years after diagnosis, ranging from 6 months to 19 years. In G2, 11 patients were evaluated, of which G2b (chronic cardiac phase) was the largest subgroup with 7 (63.6%) patients. From the G1 category, *T. cruzi* lineage identification was performed in only 56(83.6%) patients, of whom the majority, 54/56 (96.4%), had TcIV. Regarding comorbidities, these were observed in 20 (25.6%) patients, with systemic arterial hypertension (SAH) being the most prevalent (17; 85.0%). Most of the comorbities (14; 70.0%) were among individuals in the G1b category (Table 1). The control group included 20 healthy individuals with a mean age of 37 ± 13,4 years old, and 60% were male patients.

**Table 1:** Socio-epidemiological and clinical characteristics of patients with Chagas disease.

Variables	Total	G1a	G1b	G2a	G2b	P
	(n = 78)^1^	(n = 14)^1^	(n = 53)^1^	(n = 4)^1^	(n = 7)^1^	
Age, mean (SD), years	43.5 ± 16.4	40.2 ± 14.7	42.8 ± 17.3	49.5 ± 8.4	51.9 ± 15.2	0.511^2^
Sex						
Male	43 (55.1)	12 (85.7)	25 (47.2)	3 (75.0)	3 (42.9)	0.052^3^
Female	35 (44.9)	2 (14.3)	28 (52.8)	1 (25.0)	4 (57.1)	
Case Type						< 0.001^3^
Chronic	11 (14.1)	–	–	4 (100)	7 (100)	
Isolated	9 (11.5)	2 (14.3)	7 (13.2)	–	–	
Outbreak	58 (74.3)	12 (85.7)	46 (86.8)	–	–	
Follow-up time (years)	5.9 ± 5.2	–	7.4 ± 4.9	6.5 ± 2.1	6.6 ± 5.2	0.282^3^
DTU *T. cruzi*	**n = 56 (71.8)**	**n = 13 (92.8)**	**n = 43 (81.1)**	**n = 0**	**n = 0**	0.731^3^
TcI	1 (1.8)	–	1 (2.3)	–	–	
TcIV	54 (96.4)	13 (100)	41 (95.4)	–	–	
Z3 (TcIII/TcIV)	1 (1.8)	–	1 (2.3)	–	–	
Comorbidities	**n = 20 (25.6)**	**n = 2 (14.3)**	**n = 14 (26.4)**	**n = 0**	**n = 4 (57.1)**	**0.129** ^3^
Hypertension	13 (65.0)	1 (50.0)	8 (57.2)	–	4 (100.0)	
Diabetes mellitus	1 (5.0)	1 (50.0)	–	–	–	
SAH/DM	4 (20.0)	–	4 (28.6)	–	–	
Asthma	1 (5.0)	–	1 (7.1)	–	–	
Shehann Syndrome	1 (5.0)	–	1 (7.1)	–	–	

*DM – Diabetes mellitus. SAH – Systemic arterial hypertension. ^1^n (%); ^2^ANOVA; ^3^Pearson chi-square test.

### Cardiac alterations

All patients underwent an ECG, and 25 (32%) showed some type of abnormality, including bundle branch blocks and ST/T segment changes; 48 (61.5%) had an echocardiogram, and 5 (10%) exhibited abnormalities; 51 (65.4%) were submitted to 24-hour Holter monitoring, and 6 (11.8%) showed abnormalities (Table 2).

**Table 2:** Presence of cardiac alterations in patients with Chagas disease in the Amazon.

Parameters	Total (n = 78)	G1a (n = 14)¹	G1b (n = 53)^1^	G2a (n = 4)^1^	G2b (n = 7)^1^	P^2^
**Eletrocardiogram**						**0.029**
**Changed** (%)	**n = 25 (32)**	**n = 5 (35.7)**	**n = 13 (24.5)**	**–**	**n = 7 (100)**	
Electrical inactive area	3 (12)	0	2 (15.4)	0	1 (16.6)	
Primary STT changes	4 (16)	2 (40)	2 (15.4)	0	0	
First-degree atrioventricular block	1 (4)	1 (20)	0	0	0	
1º AVB + VRC	1 (4)	0	1 (7.7)	0	0	
LABD	1 (4)	0	1 (7.7)	0	0	
LABD + Sinus bradycardia	1 (4)	0	0	0	1 (16.6)	
RBBB	1 (4)	0	0	0	1 (16.6)	
iRBBB	2 (8)	0	2 (15.4)	0	0	
iRBBB + LABD	2 (8)		2 (15.4)		0	
iRBBB + Sinus bradycardia	1 (4)	0	0	0	1 (16.6)	
VES	2(8)	0	0	0	2 (28.6)	
LAO + VA	1 (4)	1 (20)	0	0	0	
AVO + iRBBB	1 (4)	0	1 (7.7)	0	0	
LVO + VA	2 (8)	1 (20)	1 (7.7)	0	0	
Wolff-Parkinson-White	1 (4)	0	1 (7.7)	0	0	
**Echocardiogram**	**n = 48 (61.5)**	**n = 11 (78.6)**	**n = 27 (50.9)**	**n = 4 (100)**	**n = 6 (85.7)**	**0.389**
Changed (%)	5 (10.4)	1 (9)	2 (7.4)	0	2 (33.3)	
**24-hour Holter**	**n = 51 (65.4)**	**n = 11 (78.6)**	**n = 30 (56.6)**	**n = 4 (100)**	**n = 6 (85.7)**	**0.410**
**Changed (%)**	6 (11.8)	3 (27.3)	2 (6.7)	0	1 (16.7)	
VES	4 (66.7)	1 (9)	2 (6.7)	0	1 (16.7)	
PVST	2 (33.3)	2 (18)	0	0	0	

VRC: Ventricular Repolarization Change; RBBB: right bundle branch block; LABD: Left Anterosuperior Divisional Block; iRBBB: Incomplete right bundle branch conduction disorder; VES: Ventricular Extrasystole; AVB: Atrioventricular block; AVO: Atrioventricular overload; LVO: Left ventricular overload; LAO: Left Atrial Overload; ARV: Ventricular Arrhythmias; PVST: Paroxysmal supraventricular tachycardia. ^1^n (%); ^2^ Pearson chi-square test.

### Routine additional examinations

From the routine laboratory tests performed, no patient presented anemia. However, there was a statistically significant difference in platelet count, leukocytes, glucose, glycated hemoglobin (HbA1c), high-density lipoprotein cholesterol (HDL), aspartate aminotransferase (AST), alanine aminotransferase (ALT), gamma-glutamyl transferase (GGT), and creatine kinase (CK) (all p < 0.05) Blood glucose and HbA1c levels were elevated in group G1b, with averages of 115 ± 66.3 and 6.3 ± 2.3, respectively. In the type 1 urine analysis, samples were collected from 44 (56.4%) patients, of whom 5 (11.4%) exhibited microscopic hematuria with more than 3 red blood cells per field. In these cases, an analysis was conducted to detect erythrocyte dysmorphisms in isolated urine samples, all of which yielded negative results. Neither proteinuria nor cylindruria was observed in any of the patients (S1 Table). A renal ultrasound imaging exam was performed in 39 (50%) patients, and none showed structural abnormalities.

### Renal and endothelial biomarkers

The estimated glomerular filtration rate (eGFR) calculated using serum creatinine (ml/min/1.73 m^2^), based on the CKD-EPI equation (Chronic Kidney Disease Epidemiology Collaboration), remained within normal values in all analyzed groups, with a mean of 109.4 ± 10.2 mL/min/1.73 m^2^. Seven out of 78 (9.0%) patients exhibited hyperfiltration with an eGFR > 120 mL/min/1.73 m^2^, of whom 2 had comorbidities (hypertension/diabetes mellitus) and one had Chagas’ cardiomyopathy. The 24-hour proteinuria samples, in turn, were within the considered normal range (Table 3).

**Table 3:** Profile of renal markers of patients.

Variables	Control (n = 20)^1^	G1a (n = 14)^1^	G1b (n = 53)^1^	G2a (n = 4)^1^	G2b (n = 7)^1^	P
**Biomarkers of kidney function**						
eGFR > 120	0 (NA%)	2 (14.3)	3 (5.7)	1 (25)	1 (14.3)	0.441^2^
Creatinine (mg/dL)	NA ± NA	0.9 ± 0.1	0.8 ± 0.2	1.0 ± 0.1	0.9 ± 0.2	0.577^3^
Urea	NA ± NA	32.8 ± 6.9	32.8 ± 10.8	29.7 ± 7.2	33.3 ± 7.8	0.953^3^
eGFR-Cr (mL/min/1.73m²)	NA ± NA	109.4 ± 10.2	101.9 ± 19.6	95.3 ± 15.3	95.2 ± 12.7	0.342^3^
24h protein (g/24h)	NA ± NA	93.1 ± 56	55.6 ± 31.3	72.4 ± 44.2	68.8 ± 20.6	0.170^3^

eGFR – Estimated glomerular filtration rate. eGFR-Cr – Estimated glomerular filtration rate for createnine. ^1^n(%); ^2^Pearson’s chi-square test; ^3^ANOVA test.

**Fig 1:**
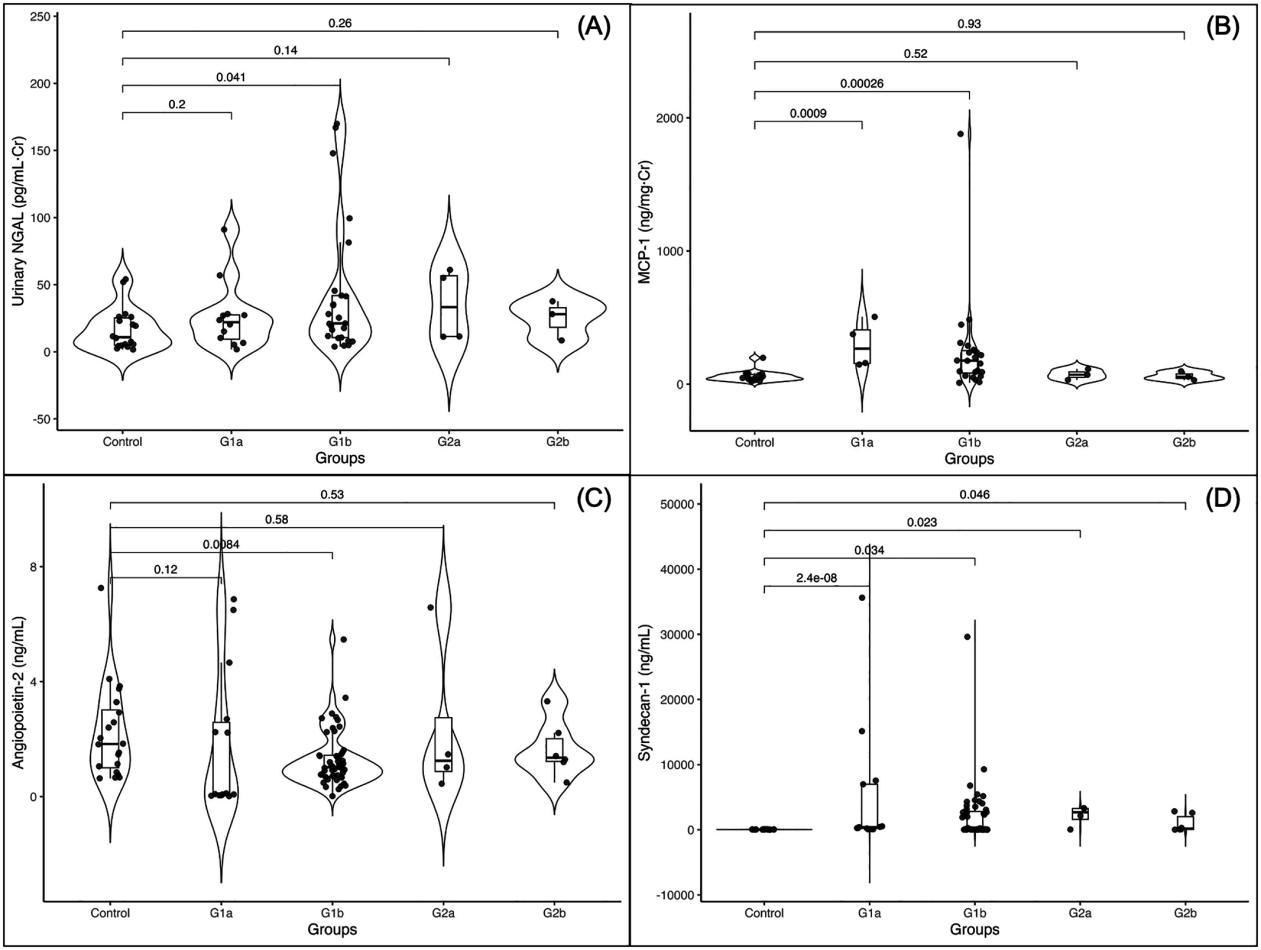
Comparison between kidney injury biomarkers (A) urinary NGAL, (B) MCP-1 and biomarkers of endothelial injury (C) ANG-2 and (D) SYN-1. Mann-Whitney U test.

The violin plots revealed significant differences in biomarker levels among the studied groups. Urinary NGAL levels were significantly higher in the G1b group compared to the Control group (p = 0.041), However, in group G2b, although all patients presented elevated NGAL levels, only three of them presented hypertension, which could suggest a possible association between elevated NGAL and hypertension in this group. However, this association was not statistically significant (p = 0.26). Furthermore, one of the hypertensive patients presented an NGAL value similar to the median of the Control group, indicating variability and limiting the strength of this association, while MCP-1 concentrations were significantly increased in both G1a and G1b relative to the Control group (p = 0.0009 and p = 0.00026, respectively). Angiopoietin-2 exhibited substantial intra-group variability, with a significant difference observed only between the G1a and G1b subgroups (p = 0.0084). All patient groups showed marked elevation in Syn-1 levels compared relative to the Control group, suggesting endothelial damage in CD patients. Overall, inflammatory and endothelial injury biomarkers were predominantly elevated in group G1, particularly in the G1b subgroup, whereas the G2 groups displayed profiles comparable to the Control group, suggesting a more severe inflammatory and tissue injury status in G1 individuals (Fig 1).

### Comorbidities

Comparing patients with comorbidities (n = 20) to those without comorbidities (n = 58), Syn-1 levels differences were not statistically significant between these groups. ANG-2 remained within normal range among those without comorbidities but slightly elevated in those with comorbidities, suggesting a potential association between ANG-2 expression and underlying pathological conditions. Patients with hypertension (SAH) or combined SAH and diabetes mellitus (DM) exhibited elevated ANG-2 levels in comparison to patients without comorbidities who had lower median ANG-2 levels. Differences in urinary NGAL adjusted for creatinine between patients with and without comorbidities did not reach statistical significance. Urinary MCP-1 remained similar between the two groups (Table 4).

**Table 4:** Profile of biomarkers of kidney and endothelial injury in the presence of comorbidities.

Variables	Comorbidities				No comorbidities	p-value^2^
	Total (n = 20)^1^	SAH (n = 13)^1^	DM (n = 1)^1^	SAH/DM (n = 4)^1^	Total (n = 58)^1^	
**Renal biomarkers**						
NGAL corrected (pg/mL-Cr)	19.5 [9.0–55.9]	14.1 [6.8–29.1]	64.1	43.9 [12.6–75.2]	28.9 [10.9–50.3]	0.871^*^
MCP-1 corrected (ng/mg-Cr)	0.15 [0.04–0.3]	0.06 [0.03–0.20]	–	0.6 [0.3–0.8]	0.14 [0.1–0.3]	0.894^*^
**Endothelial biomarkers**						
Syn-1 (ng/mL)	2015.7 [44.2–3537.2]	2357.5 [59.1–3763.2]	402.3	1790.7 [40.4–3625.4]	94.8 [34.9–2715.8]	0.262^*^
ANG-2 (ng/mL)	1.3 [1.0–2.4]	1.2 [1.0–2.4]	0.42	1.5 [1.3–3.5]	0.9 [0.5–1.5]	0.040^*^

The data are presented as medians [interquartile ranges]. ^1^n (%); ^2^Mann-Whitney U test. *Comparison between with and without comorbidities.

### Relationship between the novel biomarkers and conventional renal markers

Renal function markers – including creatinine, urea, 24-hour proteinuria and GFR- were analyzed alongside serum levels of SYN-1, ANG-2, MCP-1, and urinary NGAL. No significant correlations were observed between creatinine or urea and any of the measured biomarkers (S1 Fig, S2 Fig). Although 24-hour proteinuria remained within normal ranges, no significant correlation was noted between MCP-1 and urinary NGAL (S3 Fig). GFR showed a very weak negative correlation with urinary NGAL, but no correlation with SYN-1, ANG-2, or MCP-1 (S4 Fig).

## Discussion

This study highlights the particularities of CD in the Amazon, a region with vast geographic distances, limited healthcare access, and socioeconomic barriers that hinder the adequate monitoring of patients with CD. In this area, oral transmission prevails [28–30] especially associated with the consumption of contaminated foods, such as *açaí* [31], which differentiates the local epidemiological profile from endemic regions where vector transmission is predominant. The increased severity of acute CD cases, which is believed to be associated with a higher *T. cruzi* inoculum [32], may influence both clinical outcomes and immune responses, potentially affecting biomarker expression and the overall progression of the disease. Neurological and cardiac changes can occur in acute and chronic cases, where they tend to be fewer and milder than in other regions [33–35].

In Amazonas state, a pioneering study evaluated patients by means of cardiovascular magnetic resonance imaging, with an average follow-up of 5.2 years after diagnosis and treatment of the disease, identifying myocardial injury in 18% of cases [36]. Considering that infection by *T. cruzi* is associated with inflammatory responses affecting the cardiovascular system, both in acute and chronic cases, it is important to remember that this inflammatory process can also trigger kidney disease [11]. The presence of cardiorenal syndrome in the context of CD highlights the need for monitoring dysfunction of multiple organs [37].

Experimental studies have documented biochemical and histological alterations during the acute phase of CD, including elevated blood urea, proteinuria, kidney injury molecule-1 (KIM-1), tubular necrosis, hypercellularity, mesangial congestion, vasculitis, and fibrosis [3]. In this context, despite conventional markers of renal function, such as creatinine, urea, proteinuria, and GFR, being within normal parameters, an elevation in renal and endothelial biomarkers suggested subclinical renal and endothelial impairment in the studied population. This indicates that, even without evident clinical alterations in renal function, there are biochemical and molecular signs of renal injury and endothelial dysfunction associated with *T. cruzi* infection. The findings demonstrate greater inflammatory activation and endothelial injury in patients from the G1 group, particularly in the G1b subgroup. Increased NGAL levels suggest its potential role as an early biomarker of renal injury, while elevated MCP-1 and Syn-1 levels reinforce the presence of renal inflammation and endothelial damage, respectively, associated with Chagas disease. The variability observed in ANG-2 levels may reflect heterogeneity in the endothelial response among the studied groups.

Based on our data, elevated Syn-1 appears to be an early marker of endothelial activation in CD with increased Syn-1 levels in all phases of *T. cruzi* infection, indicating detectable endothelial dysfunction, regardless of comorbidities. However, concomitant comorbidities might intensify this dysfunction, contributing to even higher levels of this biomarker, but our results could not support this hypothesis and future studies with larger samples and longitudinal follow-up are needed to clarify this issue.

The presence of comorbidities with CD can intensify renal and cardiovascular dysfunction as demonstrated by the elevated levels of endothelial biomarkers. While the control group maintained baseline biomarker levels, indicating preserved endothelial and renal function, comorbidities in Chagas disease patients led to higher biomarker levels and greater organ impairment. Infection with *T. cruzi* alone may cause significant vascular changes, but the coexistence of additional comorbidities further worsens these effects. This scenario underscores the importance of an integrated health approach considering both *T. cruzi* infection and associated conditions that influence clinical progression and prognosis of affected individuals. The non-significant elevation of SYN-1 in patients with comorbidities could suggest a loss of endothelial glycocalyx integrity [38].

Renal injury in CD described in experimental studies and *post-mortem* arises from complex immunological, autoimmune, and inflammatory mechanisms, including direct *T. cruzi* infection of kidney cells. This leads to glomerulonephritis, cellular damage, and systemic activation of the renin-angiotensin-aldosterone system, ultimately causing both functional and structural changes in the kidneys [4,5,9,10,39,40]. These mechanisms can culminate in tubular injury, glomerulonephritis, and fibrosis, as demonstrated in experimental models [3,11,41–45].

Urinary SYN-1 levels tend to rise as acute kidney injury progresses emphasizing its significant role in both diagnosis and prognosis. Understanding the link between SYN-1 expression and renal function can provide valuable insights into the underlying mechanisms of kidney injury, potentially guiding targeted therapies [14]. This suggests that SYN-1 has the potential of an early biomarker not only for assessing the severity of renal damage, but also for evaluating cardiac functions and other related diseases.

ANG-2, an endothelial-derived protein plays a critical role in vascular regulation and inflammation with research demonstrating its connection to the development and progression of both chronic kidney disease and cardiovascular complications [18]. ANG-2 protein associated with cardiovascular diseases, serve as an important marker of chronic kidney disease (CKD) which increases cardiovascular risk. In our study, while patients without comorbidities maintained a baseline median of 0.9 ng/mL, the presence of SAH and DM pushed concentrations to 1.2 ng/mL and 1.5 ng/mL, respectively. This finding corroborates previous studies that associate ANG-2 with endothelial dysfunction, vascular inflammation, and the progression of kidney and cardiovascular diseases. Thus, our data reinforce that increased ANG-2 may reflect a more pronounced inflammatory and vasculopathic state in patients with comorbidities, thus understanding the mechanisms could lead to targeted strategies to slow kidney injury progression [15].

MCP-1, an inflammatory urinary chemokine, was elevated in the acute pre-treatment phase, serving as an early marker of kidney injury and inflammation, even without conventional markers changes. Its elevated levels correlate with kidney damage and may accelerate diabetic nephropathy, also linked to renal insufficiency in type 2 DM [46]. The association between MCP-1 and proteinuria supports its role in monitoring silent kidney lesions. Biomarkers such as MCP-1, MDA, and SYN-1 effectively identify early renal dysfunctions in parasitic infections, indicating ongoing damage before traditional parameters change. MCP-1 and MDA have also been linked to inflammation and early renal impairment in visceral leishmaniasis [47]. In intestinal schistosomiasis, elevated urinary MCP-1 is associated with albuminuria, signaling silent renal inflammation despite treatment. These can serve as a sensitive biomarker in detecting early-stage renal injury thus allowing for prompt intervention [48].

Our study findings align with those by Oliveira et al. [47], who through renal MCP-1 and MDA also detected incipient tubuloglomerular dysfunction and renal inflammation in visceral leishmaniasis patients. This further emphasizes the importance of identifying and using novel biomarkers for early kidney injury detection in parasitic diseases. Both studies show that functional and inflammatory changes can occur before routine renal tests can detect abnormalities even though functional tests appeared normal. Further indicating that inflammation and tubuloglomerular dysfunction begin early as the infection establishes and progresses. Biomarkers like NGAL and KIM-1 can effectively identify acute kidney injury (AKI) earlier than serum creatinine. Van Wolwesinkel et al. [49] demonstrated NGAL’s strong predictive capacity for renal injury in *Plasmodium falciparum* infection, which supports our observation of elevated NGAL in acute CD.

NGAL levels were elevated in acute CD patients, group G1b, indicating renal changes linked to CD even after treatment. These findings support NGAL’s potential as a promising biomarker for early detection of renal injury in acute CD surpassing conventional tests [22]. Additionally, MCP-1 in acute CD and SYN-1 in acute and chronic CD increases reflect inflammation and endothelial dysfunction before clinical signs, enabling timely intervention.

This study found that biomarkers like creatinine and urea, might not be sufficient for early detection of lesions, highlighting the need to develop and incorporate novel biomarkers such as SYN-1, MCP-1, and NGAL, that can identify early subclinical changes in the context of CD before dysfunctions occur. Understanding such processes and mechanisms is of utmost importance in the Amazon region, more so because of acute CD predominance and difficulties in accessing medical care hinder early diagnosis and management of the disease. The complexity of these pathophysiological mechanisms underscores the need for careful monitoring and integrated approaches to identify early possible renal damage among CD patients. Indeed, our data indicate that NGAL shows significant differences mainly in treated acute infection, highlighting its potential as an early marker of renal injury at this stage. MCP-1 shows elevations predominantly in the acute phase, regardless of treatment, reinforcing its relevance in detecting early renal inflammation. SYN-1 shows an increase in different phases of the disease, suggesting its association with endothelial damage throughout the course of the disease. As for ANG-2, our results indicate that it does not rise significantly at any stage of the disease compared to the healthy control group, suggesting that its usefulness as a diagnostic or prognostic marker in CD may be limited or specific to other contexts that require further evaluation.

## Conclusion

The new biomarkers studied (NGAL, SYN-1, ANG-2, and MCP-1) in patients of the Amazon region showed an association with different phases of CD and with signs of endothelial and renal dysfunction present in the clinical phases, suggesting a potential value in the detection of subclinical changes related to the disease. The elevation of novel biomarkers in different clinical phases of CD reveals the role of inflammatory and endothelial processes in the pathogenesis of renal and cardiovascular complications, often before detected by conventional diagnostic indicators. However, longitudinal studies are necessary to confirm their usefulness in predicting the development of renal or vascular damage over time.

### Limitations of the study

This study had several limitations. There was no follow-up that could confirm changes in traditional markers of kidney injury among those with elevated novel biomarkers. The difficulty in the longitudinal monitoring of patients compromises the epidemiological and clinical assessment of disease progression in the Amazon region due to limited accessibility to healthcare services and low adherence to post-treatment follow-up, thus affecting the collection of complete and longitudinal data for deeper analysis. Finally, the low number of chronic phase patients and those with severe cardiac involvement limited a detailed analysis of these subgroups, making it difficult to generalize findings to all disease stages. Future studies with more robust longitudinal follow-up are necessary to confirm the clinical-epidemiological profile and validate the role of these biomarkers for early detection, interventions, and improved care for patients.

## Supporting information

S1 File Routine additional examinations. (DOCX)

S2 File Relationship between the new biomarkers and traditional renal markers. (DOCX)

S1 Table Profile of routine laboratory tests of patients with Chagas disease. (DOCX)

S1 Fig Pearson’s correlation (ρ) between biomarker levels and serum creatinine levels. (TIFF)

S2 Fig Spearman’s correlation (ρ) between biomarker levels and urea levels. (TIFF)

S3 Fig Spearman’s correlation (ρ) between biomarker levels and proteinuria. (TIFF)

S4 Fig Spearman’s correlation (ρ) between biomarker levels and glomerular filtration rate. (TIFF)
