# Supplementary material for: Renal and endothelial biomarkers in Chagas disease in the Brazilian Amazon region: Early indicators of kidney injury and disease progression
Source: PLoS One. 2026 Jul 17;21(7):e0353749. doi: 10.1371/journal.pone.0353749 (PMC13379015; doi:10.1371/journal.pone.0353749)
Supplement: S2 File — (DOCX) [file pone.0353749.s002.docx]

**Supporting Information**

**Relationship between the new biomarkers and traditional renal markers**

Traditional renal function markers - including creatinine, urea, 24-hour proteinuria and GFR- were analyzed alongside serum levels of SYN-1, ANG-2, MCP-1, and urinary NGAL. No significant correlations were observed between creatinine or urea and any of the measured biomarkers (S1 Fig, S2 Fig). Although 24-hour proteinuria remained within normal ranges, a weak, non-significant positive correlation was noted between MCP-1 and urinary NGAL (S3 Fig). GFR showed a very weak negative correlation with urinary NGAL, but no correlation with SYN-1, ANG-2, or MCP-1 (S4 Fig). These findings suggest potential subclinical alterations in renal or endothelial function that are not detectable by conventional renal markers.


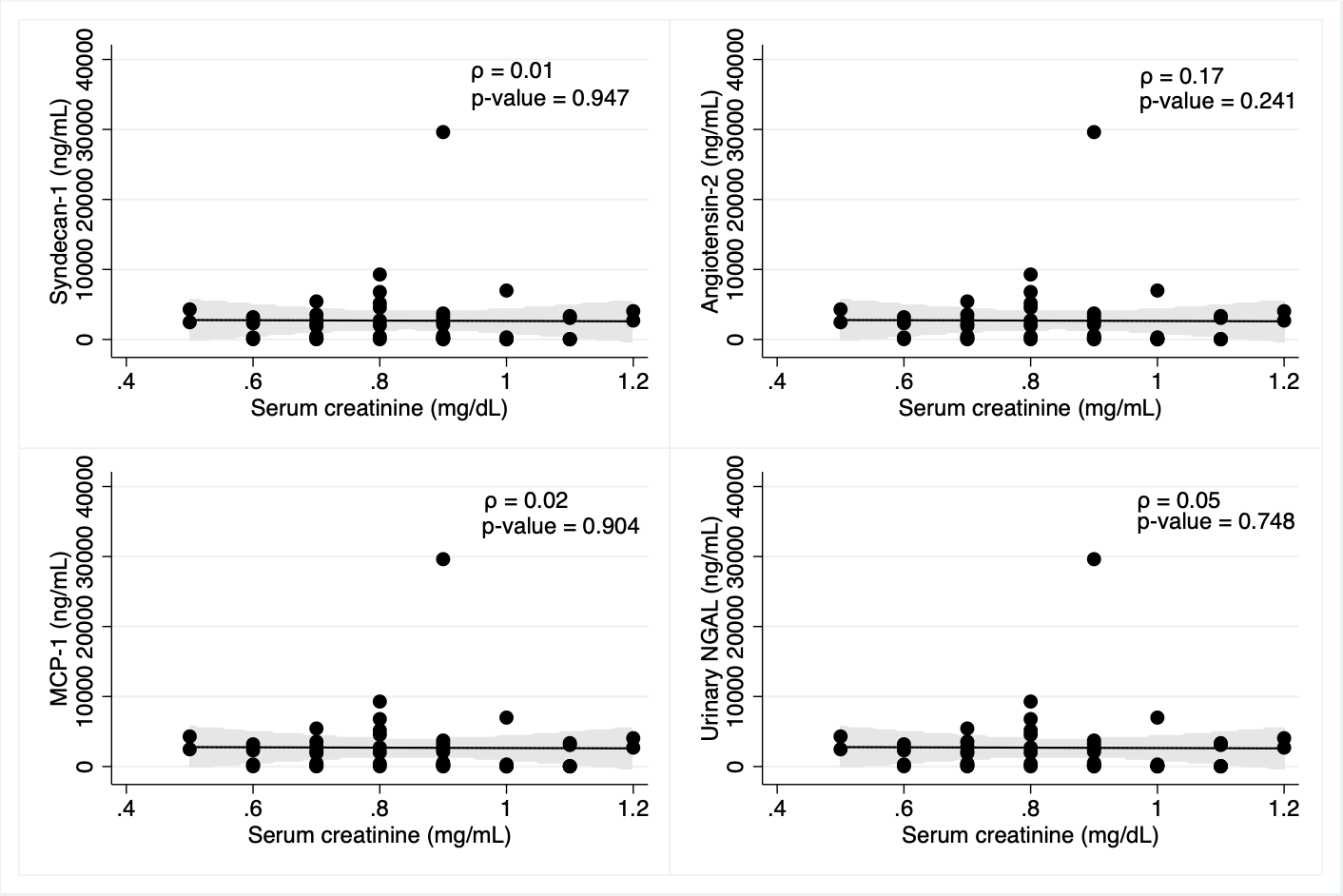


**S1 Fig. Pearson's correlation (ρ) between biomarker levels and serum creatinine levels.**


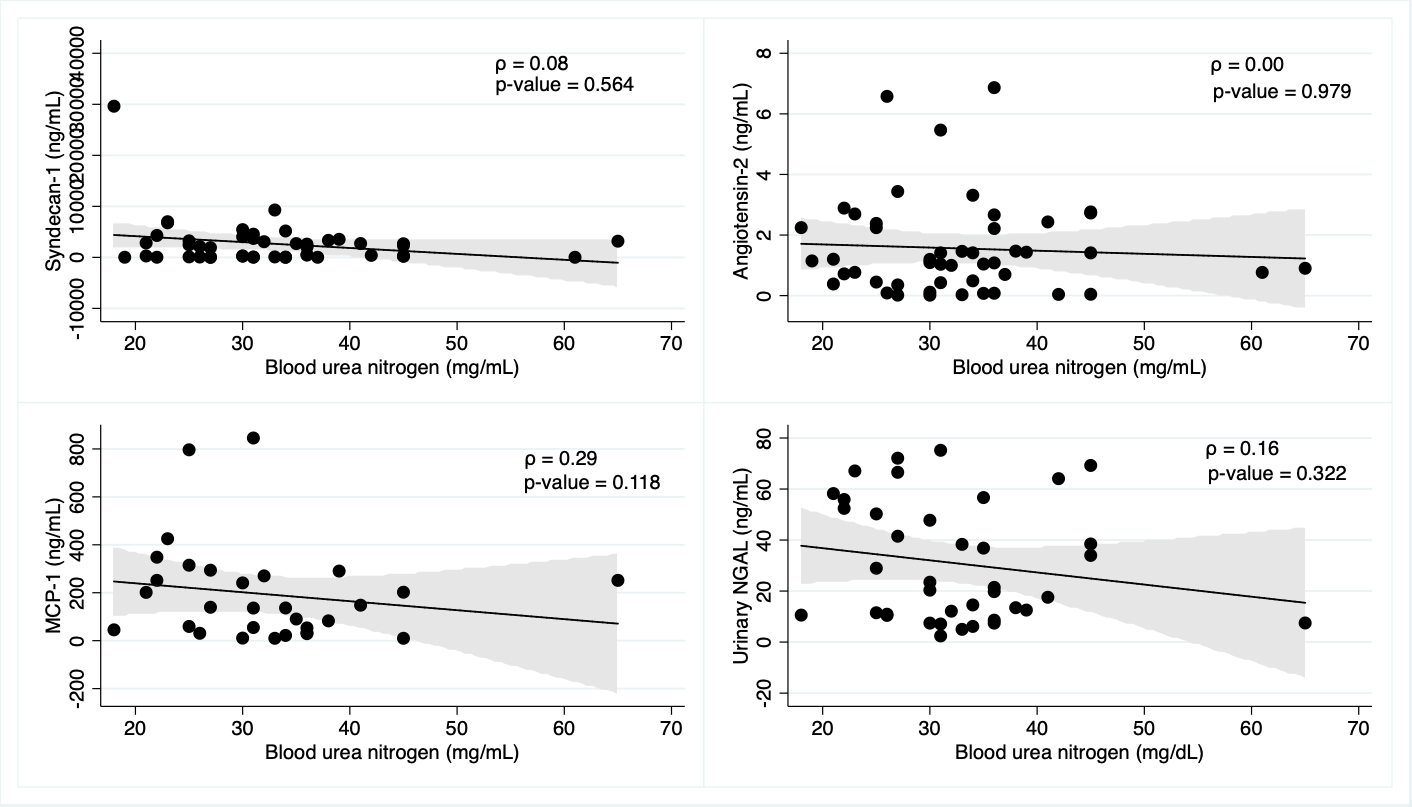


**S2 Fig. Spearman's correlation (ρ) between biomarker levels and urea levels**


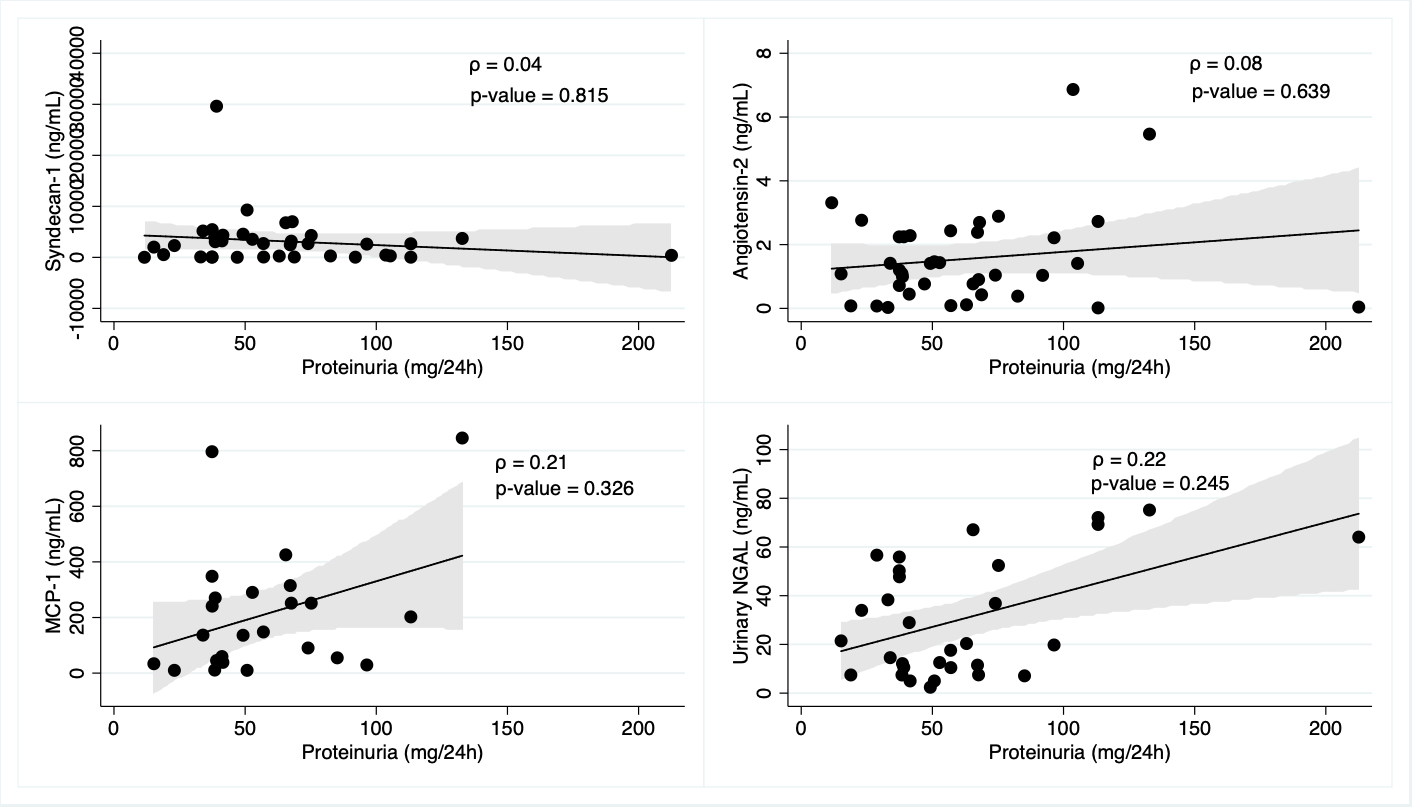


**S3 Fig. Spearman's correlation (ρ) between biomarker levels and proteinuria.**


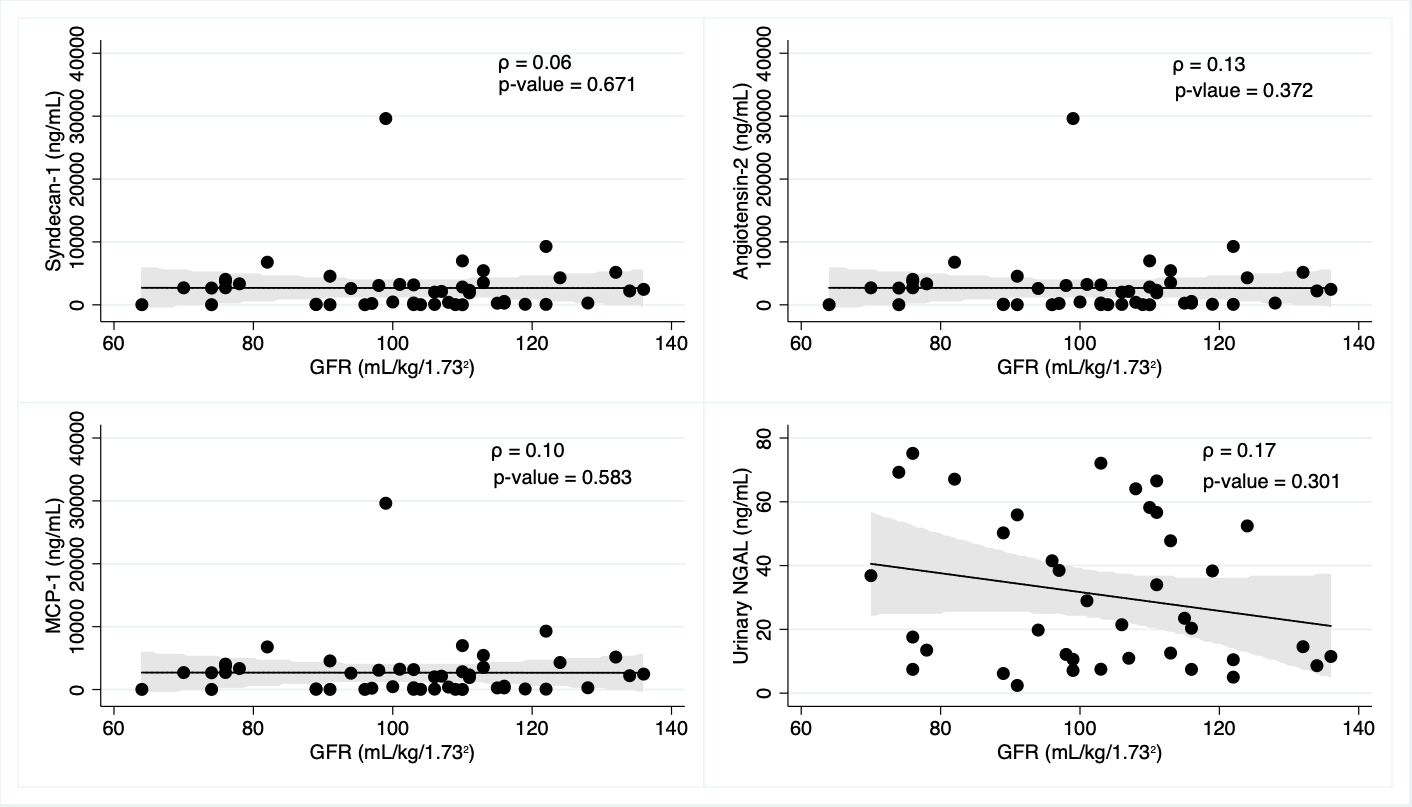


**S4 Fig. Spearman's correlation (ρ) between biomarker levels and glomerular filtration rate.**
