# Supplementary material for: Renal and endothelial biomarkers in Chagas disease in the Brazilian Amazon region: Early indicators of kidney injury and disease progression
Source: PLoS One. 2026 Jul 17;21(7):e0353749. doi: 10.1371/journal.pone.0353749 (PMC13379015; doi:10.1371/journal.pone.0353749)
Supplement: S1 Table — (DOCX) [file pone.0353749.s003.docx]

**Supporting Information**

**Routine additional examinations**

From the routine laboratory tests performed, no patient presented anemia. However, there was a statistically significant difference in platelet count, leukocytes, glucose, glycated hemoglobin (HbA1c), high-density lipoprotein cholesterol (HDL), aspartate aminotransferase (AST), alanine aminotransferase (ALT), gamma-glutamyl transferase (GGT), and creatine kinase (CK) (all p<0.05) Blood glucose and HbA1c levels were elevated in group G1b, with averages of 115 ± 66.3 and 6.3 ± 2.3, respectively. In the type 1 urine analysis, samples were collected from 44 (56.4%) patients, of whom 5 (11.4%) exhibited microscopic hematuria with more than 3 red blood cells per field. In these cases, an analysis was conducted to detect erythrocyte dysmorphisms in isolated urine samples, all of which yielded negative results. Neither proteinuria nor cylindruria was observed in any of the patients (S1 Table). A renal ultrasound imaging exam was performed in 39 (50%) patients, and none showed structural abnormalities.

**S1 Table. Profile of routine laboratory tests of patients with Chagas disease**

| Variables | Total | G1a | G1b | G2a | G2b | P |
| --- | --- | --- | --- | --- | --- | --- |
|  | **(n=78)¹** | **(n=14)¹** | **(n=53)¹** | **(n=4)¹** | **(n=7)¹** |  |
| Hemogram |  |  |  |  |  |  |
| Hemoglobin (g/dL) | 14.3 ± 1.7 | 13.2 ± 1.4 | 14.4 ± 1.6 | 16.4 ± 0.3 | 15 ± 1.2 | 0.534^2^ |
| Hematocrit (%) | 43.4 ± 5.0 | 39.9 ± 4.2 | 43.6 ± 4.3 | 49.1 ± 0.2 | 46.8 ± 6.4 | 0.107^2^ |
| Platelets (/10^3^) | 261 ± 11 | 323 ± 19 | 252 ± 59 | 225 ± 53 | 208 ± 64 | < 0.001^2^ |
| Leucocytes (/10^3^) | 7.4 ± 2.5 | 7.9 ± 2.3 | 7.6 ± 2.4 | 4.6 ± 5.8 | 6.2 ± 8 | 0.048^2^ |
| Biochemistry tests |  |  |  |  |  |  |
| Glucose (g/dL) | 108.9 ± 52.6 | 101.7 ± 15.6 | 115 ± 66.3 | 96 ± 2.6 | 97.6 ± 6.2 | < 0.001^2^ |
| HbA1c (%) | 6.1 ± 2.0 | - | 6.3 ± 2.3 | 5.6 ± 0.2 | 5.4 ± 0.1 | < 0.001^2^ |
| Cholesterol total (mg/dL) | 192.1 ± 42.4 | 162.9 ± 21.5 | 200 ± 47.2 | 188 ± 26.6 | 198.7 ± 31.8 | 0.076^2^ |
| LDL-cholesterol (mg/dL) | 122.5 ± 37 | 97.7 ± 23.5 | 131 ± 40.6 | 117 ± 14 | 123.5 ± 28.1 | 0.137^2^ |
| HDL-cholesterol (mg/dL) | 42.3 ± 22.3 | 28.9 ± 7.6 | 41.7 ± 9.8 | 51 ± 29.3 | 62.8 ± 52.6 | < 0.001^2^ |
| Triglycerides (mg/dL) | 156.8 ± 86.1 | 182 ± 105.2 | 146 ± 84.6 | 127 ± 53.4 | 178.8 ± 74.3 | 0.666^2^ |
| AST (U/L) | 35.6 ± 33.0 | 63.5 ± 52.3 | 28.6 ± 21.5 | 26.3 ± 3.1 | 21.2 ± 6.4 | < 0.001^2^ |
| ALT (U/L) | 45.6 ± 47.5 | 85 ± 85.6 | 35.3 ± 18.5 | 33.3 ± 11.6 | 28.4 ± 24.8 | < 0.001^2^ |
| Alkaline phosphatase (U/L) | 258 ± 106.4 | 360 ± 131.3 | 227 ± 62.8 | 227.8 ± 62.8 | 203.3 ± 63.5 | 0.038^2^ |
| Gamma GT (U/L) | 55.6 ± 52.7 | 108.2 ± 78.1 | 37.1 ± 16.3 | 27.5 ± 14.8 | 35.3 ± 26.3 | < 0.001^2^ |
| Albumin (g/dL) | 4.4 ± 0.3 | 4.1 ± 0.3 | 4.6 ± 0.2 | 4.3 ± 0.4 | 4.5 ± 0.5 | 0.469^2^ |
| Creatine kinase (U/L) | 143 ± 321.1 | 46.8 ± 13.4 | 96.8 ± 40.6 | **-** | 552 ± 820.6 | < 0.001^2^ |
| Potassium (mEq/L) | 4.4 ± 0.4 | 4.4 ± 0.5 | 4.4 ± 0.4 | 4.4 ± 0.9 | 4.3 ± 0.3 | 0.135^2^ |
| Sodium (mEq/L) | 139 ± 3.4 | 138.3 ± 3.7 | 139.2 ± 3.6 | 140.7 ± 2.5 | 138.3 ± 2.7 | 0.822^2^ |
| Calcium (mg/dL) | 10.1 ± 0.6 | 9.8 ± 1.2 | 10.3 ± 0.4 | **-** | 9.9 ± 0.6 | 0.395^2^ |
| Urinalysis, n (%) | **44 (56.4)** | **10 (71.4)** | **26 (49.1)** | **2 (50)** | **6 (85.7)** |  |
| Density | 1016 ± 8.0 | 1017 ± 7.5 | 1017 ± 8.1 | 1015 ± 14.1 | 1013 ± 8.2 | 0.830^2^ |
| pH | 6.1 ± 0.3 | 5.9 ± 0.3 | 6.1 ± 0.2 | 6.5 ± 0.7 | 6.1 ± 0.2 | 0.130^2^ |
| Hemoglobin | 9 (20.5) | - | 7 (26.9) | - | 2 (33.3) | 0.225^3^ |
| Glucose | 3 (6.8) | - | 2 (7.7) | - | 1 (16.7) | 0.609^3^ |
| Pyocytes | 17 (38.6) | 4 (40) | 13 (50) | - | - | 0.091^3^ |
| Red Blood Cells | 5 (11.4) | - | 4 (15.4) | - | 1 (16.7) | 0.547^3^ |
| Crystals | 10 (22.7) | 4 (40) | 6 (23.1) | - | - | 0.256^3^ |
| Bacteria | 19 (44.2) | 5 (50) | 14 (56) | - | - | 0.048^3^ |

*AST –Aspartate aminotransferase. ALT – Alanine aminotransferase. Gamma GT - Gamma glutamyltransferase . HbA1c – Glycated hemoglobin .* ^1^n (%); ^2^ANOVA; ^3^ Pearson chi-square test
