# Supplementary figures and images for: Renal and endothelial biomarkers in Chagas disease in the Brazilian Amazon region: Early indicators of kidney injury and disease progression
Source: PLoS One. 2026 Jul 17;21(7):e0353749. doi: 10.1371/journal.pone.0353749 (PMC13379015; doi:10.1371/journal.pone.0353749)

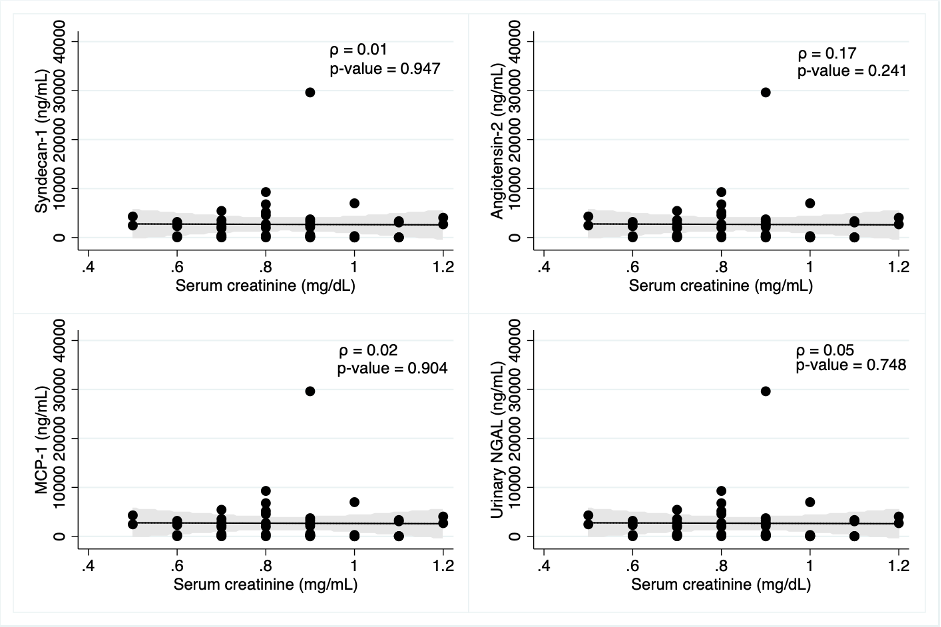

Supplement: S1 Fig — (TIFF) [file pone.0353749.s004.tiff]

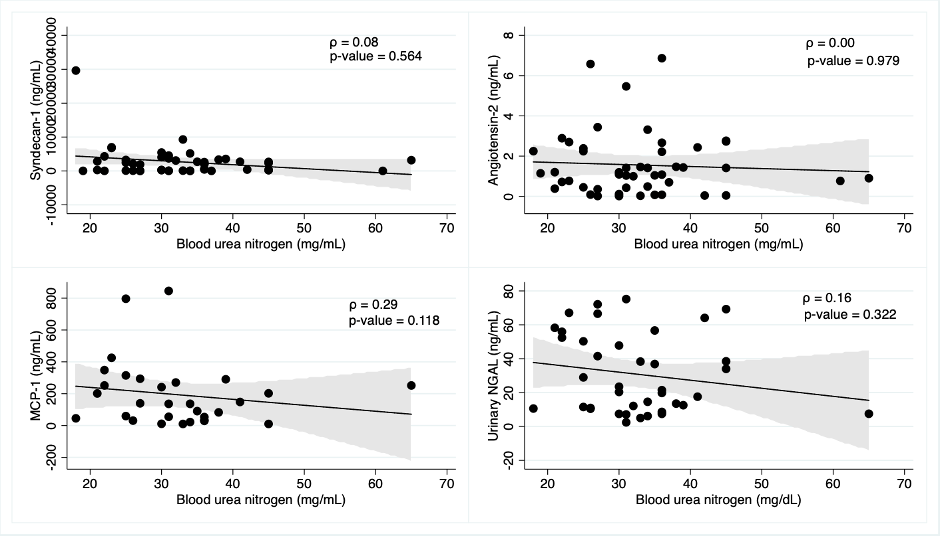

Supplement: S2 Fig — (TIFF) [file pone.0353749.s005.tiff]

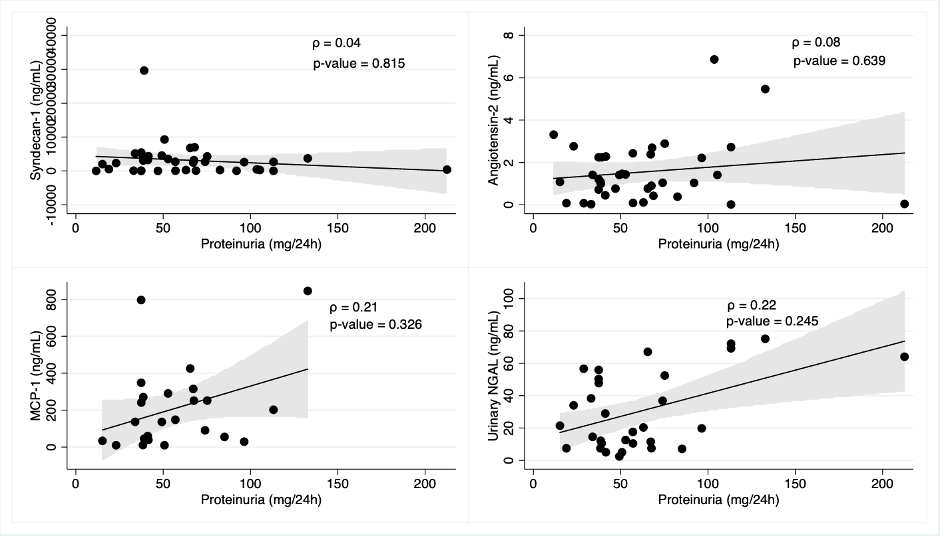

Supplement: S3 Fig — (TIFF) [file pone.0353749.s006.tiff]

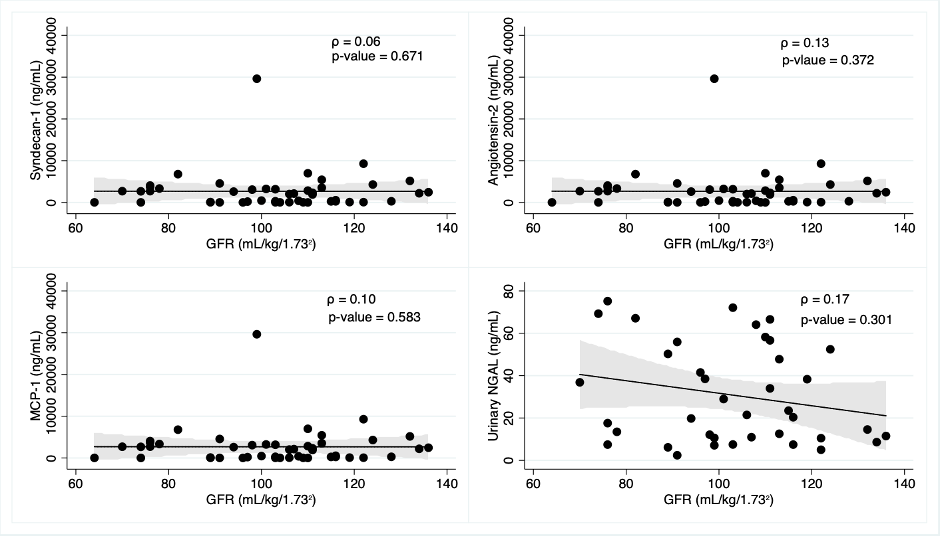

Supplement: S4 Fig — (TIFF) [file pone.0353749.s007.tiff]
